# Improvement of multisource localization of magnetic particles in an animal

**DOI:** 10.1038/s41598-021-88847-8

**Published:** 2021-05-05

**Authors:** Chin-Wei Lin, Shu-Hsien Liao, Han-Sheng Huang, Li-Min Wang, Jyh-Horng Chen, Chia-Hao Su, Kuen-Lin Chen

**Affiliations:** 1 Graduate Institute of Applied Physics, National Taiwan University, Taipei, Taiwan; 2 Institute of Electro-Optical Science and Technology, National Taiwan Normal University, Taipei, Taiwan; 3 Interdisciplinary MRI/MRS Lab, Department of Electrical Engineering, National Taiwan University, Taipei, Taiwan; 4 Institute for Translational Research in Biomedicine, Kaohsiung Chang Gung Memorial Hospital, Kaohsiung, Taiwan; 5 Department of Physics, National Chung Hsing University, Taichung, Taiwan

**Keywords:** Molecular imaging, Biomedical engineering

## Abstract

In this simulation work, the linearized Bregman iterative algorithm was applied to solve the magnetic source distribution problem of a magnetic particle imaging (MPI) system for small animals. MPI system can apply an excitation magnetic field, and the induced magnetic field from the magnetic nanoparticles (MNPs) can be detected by the sensors of MPI system. With a gaussian distribution source at the upper side of the mouse brain, sensors set above the mouse brain and the constant excitation magnetic field, the average deviation of the calculated source distribution from the multiplane scanning along the axis away from the mouse brain and the closest plane scanning are 2.78 × 10^–3^ and 2.84 × 10^–3^ respectively. The simulated result showed that combination of multiplane scanning hardly improves the accuracy of the source localization. In addition, a gradient scan method was developed that uses gradient magnetic field to scan the mouse brain. The position of the maximum of the lead field matrix will be controlled by the gradient field. With a set up gaussian distribution source at the bottom of the mouse brain, the average deviation of the calculated source distribution from the gradient scan method and the constant field are 4.42 × 10^–2^ and 5.05 × 10^–2^. The location error from the two method are 2.24 × 10^–1^ cm and 3.61 × 10^–1^ cm. The simulation showed that this method can improve the accuracy compared to constant field when the source is away from the sensor and having a potential for application.

## Introduction

Medical imaging has become a very important tool to help doctors diagnose diseases because it can provide noninvasive anatomical information of organs with advance medical technology. Many imaging tools, such as X-ray imaging, magnetic resonance imaging (MRI), computed tomography (CT), and positron emission tomography (PET), are well developed and widely used in clinical application. In order to improve the cure rate of treatment, early finding and accurately locating the lesion are very crucial issues. Therefore, improving image quality and developing new imaging methods are urgent and necessary. Recently, magnetic particle imaging (MPI) for tomography technique have been considered to have potential for medical application^1^, since MPI can provide fast dynamic 3D images of magnetic nanoparticles (MNPs) with good spatial resolution. MNPs can be biofunctionalized and connected with specific proteins to trace the specific cancer or diseases. Combination of MPI and bio-MNPs can achieve medical application^2^.

MPI mainly detects the magnetic fields generated by the magnetic tracers, such as magnetic nanoparticles and contrast agent, and then inversely calculates the magnetic field distribution from the information of the sensor field map. The whole behavior of the detection can be seen as a mathematical equation of *A* × *u* = *f*, in which *A* represents the magnetic field operator (also known as lead field matrix), *u* represents the source distribution, and *f* represents the field map^3–5^. Calculating the field map from the source distribution is called forward problem. In contrast, getting the source distribution from the field map is called inverse problem. The boundary element model (BEM) constructed from the MRI image is the calculation space, which is assumed to be homogeneous for simplicity. The arrangement of the sensors and BEM can establish the forward model directly, i.e., the lead field matrix of MPI system, based on the physical law of electromagnetism^3–9^. On the other hand, the field inverse problem is the so-called ill-posed problem because there are multiple possible solutions with the same magnetic field map^3,4^. In recent years, several techniques have been developed to solve the ill-posed problem. The regulation term is being applied to make the ill-posed problem solvable. Besides, constraints according to our experimental condition are also applied to help in obtaining the most possible solution^3^. Generally, the localization accuracy becomes worse when the magnetic source is far from the sensors. In this work, we simulated the detection of the MPI system. Besides, a “gradient scan method” was established to improve the accuracy of the calculated magnetic particle distribution, especially for the “deeper sources,” was developed.

## Results and discussion

### Constant field

**Figure 1:**
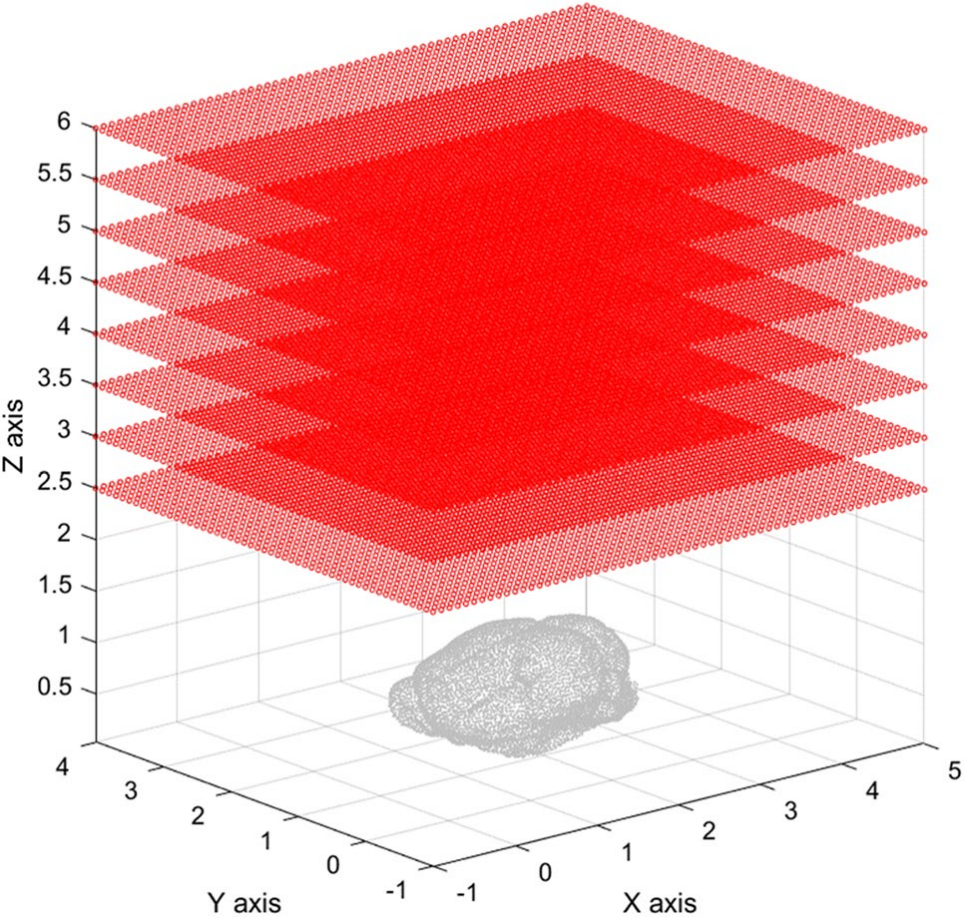
*XY* scanning plane of the sensor from *Z* = 2.5 cm to *Z* = 6.0 cm and an interval of 0.5 cm.

**Figure 2:**
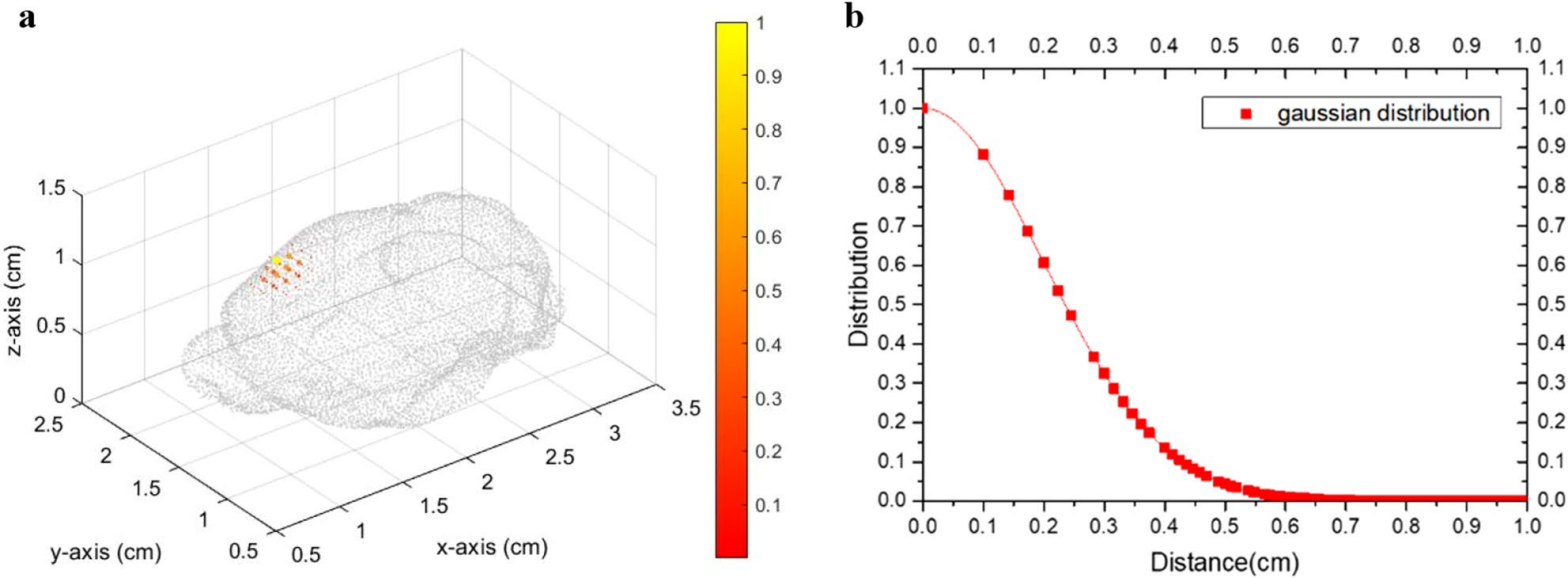
(**a**) Gaussian distribution of magnetic particles and (**b**) their distribution plot with a sigma of 0.1 cm.

The *XY* scanning plane of the sensor was still 6.0 × 5.0 cm^2^ with an interval of 0.1 cm above the mouse brain. The *Z* coordinate of the scanning plane moved away along the *Z* axis from *Z* = 2.5 cm to *Z* = 6.0 cm with an interval of 0.5 cm. The magnetic field used was 0.5 mT. The schematic diagram is shown in Fig. 1. To simulate cancer in the mouse brain, it was assumed that the magnetic particles in the cancer has a Gaussian distribution with a center at [1.5,1.8,1.0] and a sigma of 0.1 cm as shown Fig. 2.

**Figure 3:**
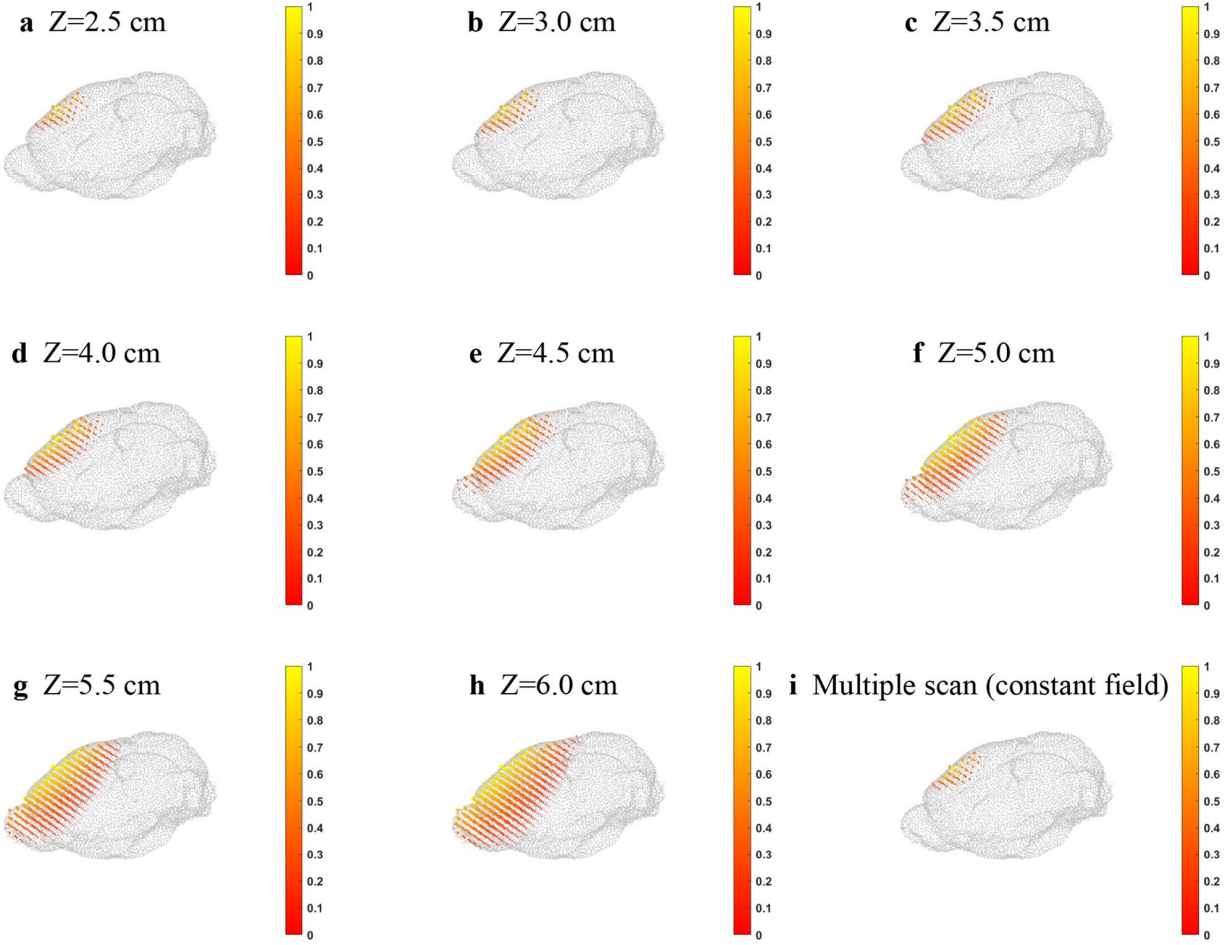
Inverse source distribution with Z position of the sensors at (**a**) 2.5 cm, (**b**) 3.0 cm, (**c**) 3.5 cm, (**d**) 4.0 cm, (**e**) 4.5 cm, (**f**) 5.0 cm, (**g**) 5.5 cm, and (**h**) 6.0 cm. (**i**) The combination of all scanning planes with different Z positions (multiple scan).

The results of all the inverse distributions with different *Z* positions of the sensors are shown in Fig. 3. Besides, all the *XY* scanning with different *Z* positions were combined into a lead field matrix and then applied them into the algorithm to solve the inverse problem. We called scanning with different *Z* positions the “multiple scan.” Fig. 3 shows that the inverse distribution is getting disperse when the *Z* position of the sensor is larger.

The average deviation and location error of the inverse distribution with different *Z* positions of the sensor and multiple scan is shown in Table 1. The deviation results agree with the distribution results in Fig. 3. If the sensor is far from the source, the inverse distribution calculated from the algorithm will become worse. The location error represents the position difference of the maximum distribution. When the *Z* position of the sensor was 5.5 cm, the location error started to be nonzero.

**Table 1:** Location error of the inverse distribution with different *Z* positions.

Scanning plane position	Max distribution position			Location error	Average deviation
	*X*	*Y*	*Z*		
Original position	1.5	1.8	1.0	–	–
2.5 cm	1.5	1.8	1.0	0	0.00278
3.0 cm	1.5	1.8	1.0	0	0.00335
3.5 cm	1.5	1.8	1.0	0	0.00382
4.0 cm	1.5	1.8	1.0	0	0.00427
4.5 cm	1.5	1.8	1.0	0	0.00481
5.0 cm	1.5	1.8	1.0	0	0.00529
5.5 cm	1.6	2.0	0.9	0.2449	0.00566
6.0 cm	1.6	2.0	0.9	0.2449	0.00588
A multiple scan	1.5	1.8	1.0	0	0.00284

The calculated inverse distribution of the multiple scan did not seem to have a significant improvement compared to the inverse distribution for the smaller *Z* position such as *Z* = 2.5 or 3.0 cm. The scanning plane with larger *Z* position values did not help. The scanning plane with *Z* = 2.5 cm was the closest scanning plane from the BEM. It was the most influential scanning plane among the others in the combination of the lead field matrix. The inverse calculation was mainly dependent on the closet scanning plane, and the other planes had minimal contribution. The sensor just needed to be set up as close as possible to the source to have a good inverse source distribution.

### Gradient scan method

#### Single-source Simulation

##### Square BEM

**Figure 4:**
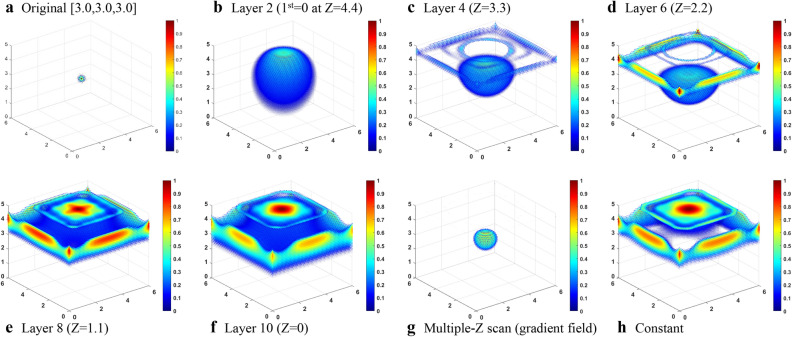
(**a**) Original distribution. Inverse distribution with the gradient field that made the lead field matrix to have local maximum at different layers: (**b**) layer 2 (*Z* = 4.4 cm), (**c**) layer 4 (*Z* = 3.3 cm), (**d**) layer 6 (*Z* = 2.2 cm), (**e**) layer 8 (*Z* = 1.1 cm), and (**f**) layer 10 (*Z* = 0 cm). (**g**) Ten-layer multiple *Z* scan (gradient field) and (**h**) constant field.

In this simulation, the *XY* scanning plane of the sensor measured 8.0 × 8.0 cm^2^ with a scanning interval of 0.5 cm. The BEM was a 6.0 × 6.0 × 5.0-cm^3^ cuboid with an interval of 0.1 cm. The *Z* position of the scanning plane was fixed at *Z* = 6.0 cm, and the bias field was 0.5 mT. The original source was a Gaussian distribution with the center at [3.0,3.0,3.0] and a sigma of 0.1 cm as shown in Fig. 4a. The gradient fields were applied to make the lead field matrix to have local maximum at different layers. There are 10 layers with different Z positions from top to the bottom of the BEM and the calculated results of the even layers were demonstrated.

In Fig. 4b, the inverse source distribution from the maximum lead field at layer 2 was higher than the original source. When the *Z* position of the layer at which we applied gradient field had a maximum value in the lead field matrix that was close to the position of the setup source, the inverse distribution became better as shown in Fig. 4c,d. In this case, the generated maximum was not the largest magnitude of the lead field matrix but because of its sign. The absolute value of the lead field at the higher position of the BEM increased when the Z position of the layer became lower. The weight of the topside of the BEM increased dramatically in the lead field matrix, so there were fake distributions at the topside of the BEM in the inverse calculation as shown in Fig. 4d–f.

Figure 4g is the inverse calculation of the multilayer scanning that integrates the data of all 10 layers. We called multilayer scanning with gradient field the “multiple *Z* scan.” The result showed good similarity with the original source, and it can be seen that the results calculated from the constant field were not close to the original source as shown in Fig. 4h. Therefore, the idea on making the local maximum of the lead field matrix was feasible. This method used the gradient field to scan the BEM, and inverse results calculated from the combination of the all the lead field matrices with different gradient fields were even better than those of the single gradient field with maximum lead field close to the position of the source.

##### Mouse brain BEM

The method of the gradient field multiple *Z* scan on the mouse brain BEM was then applied. The *XY* scanning plane of the sensor was 6.0 × 5.0 cm^2^ with a scanning interval of 0.5 cm. The *Z* position of the scanning plane was fixed at *Z* = 2.5 cm. The setup source distribution was normal distribution with sigma equal to 0.1 cm and center at [2.0,1.0,1.1], [2.0,1.0,0.6], and [2.0,1.0,0.1]. The bias field was 0.5 mT. The number of total divided layers was 5. Besides, we took a threshold value of 0.3 to make the image concise.

**Figure 5:**
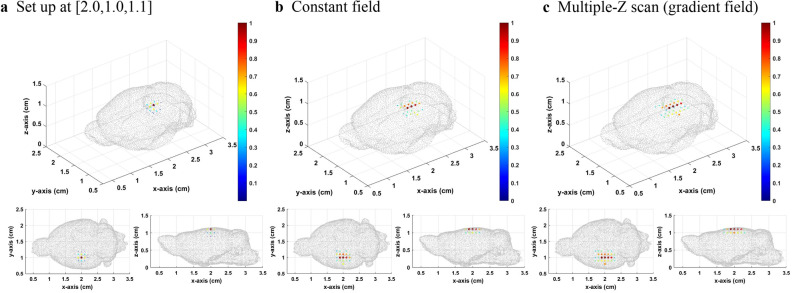
(**a**) The original source distribution center at [2.0,1.0,1.1], (**b**) inverse distribution calculated from the lead field matrix of MPI system with the constant field, and **c** inverse distribution results from the multiple *Z* scan (gradient field) with thresholds equal to 0.3.

**Figure 6:**
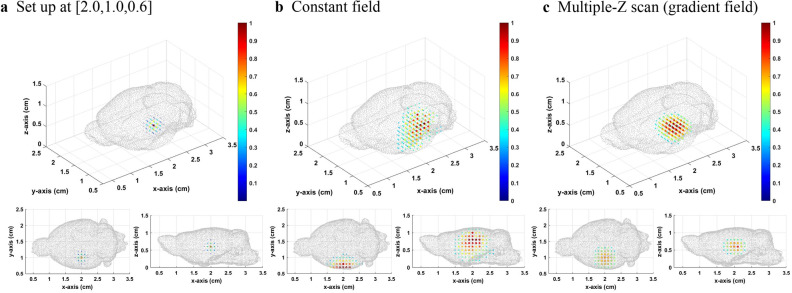
(**a**) The original source distribution center at [2.0,1.0,0.6], (**b**) inverse distribution calculated from the lead field matrix of MPI system with the constant field, and c inverse distribution result from the multiple Z scan (gradient field) with thresholds equal to 0.3.

**Figure 7:**
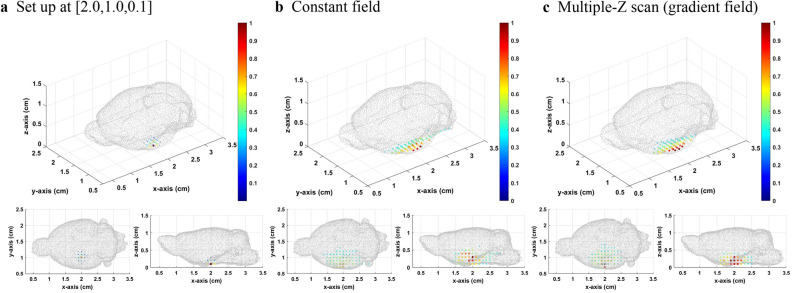
(**a**) The original source distribution center at [2.0,1.0,0.1], (**b**) inverse distribution calculated from the lead field matrix of MPI system with the constant field, and c inverse distribution result from the multiple Z scan (gradient field) with thresholds equal to 0.3.

**Figure 8:**
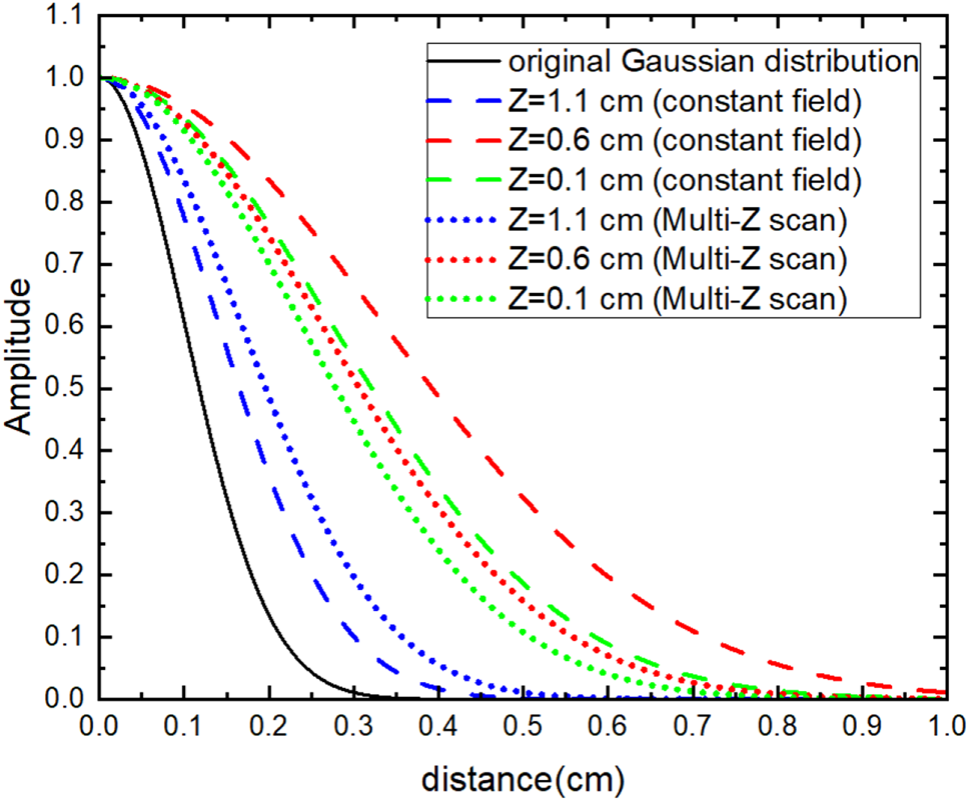
Fitting curve of the normal distribution of the source.

From Figs. 5, 6, 7, it can be seen that when the source is close to the sensor, the results of the constant field and gradient field multiple *Z* scan both have a good inverse solution similar with the those of the original distribution. Because the positions closed to the sensors had good diversity in corresponding lead field matrix, the result of the constant field was better for sensing the source very closed to sensors. When the distance between sensor and source increases, the results of the constant field and gradient field multiple *Z* scan both become worse. However, the deterioration of the inverse result of the constant field was worse than that of the gradient field multiple *Z* scan. The Gaussian fitting curve in Fig. 8 and the average deviation and location error in Table 2 all have the same trends. When the source was at the bottom, the average deviation, location error and gaussian fitting of both constant field and gradient field multiple *Z* scan became better due to the boundary of the bottom of the BEM.

**Table 2:** Location error and average deviation of the inverse distribution of the constant and gradient field multiple *Z* scan.

	Original source position	Calculated maximum distribution position	Location error (cm)	Average deviation
Constant field	[2.0, 1.0, 0.1]	[2.0, 0.7, 0.3]	0.361	0.00505
	[2.0, 1.0, 0.6]	[2.0, 0.7, 0.8]	0.361	0.00984
	[2.0, 1.0, 1.1]	[2.0, 1.0, 1.1]	0.000	0.00215
Multiple Z scan (gradient)	[2.0, 1.0, 0.1]	[2.0, 0.8, 0.2]	0.224	0.00442
	[2.0, 1.0, 0.6]	[2.0,1.0, 0.6]	0.000	0.00664
	[2.0, 1.0, 1.1]	[2.0, 1.0, 1.1]	0.000	0.00287

#### Multisource simulation

##### Square BEM

**Figure 9:**
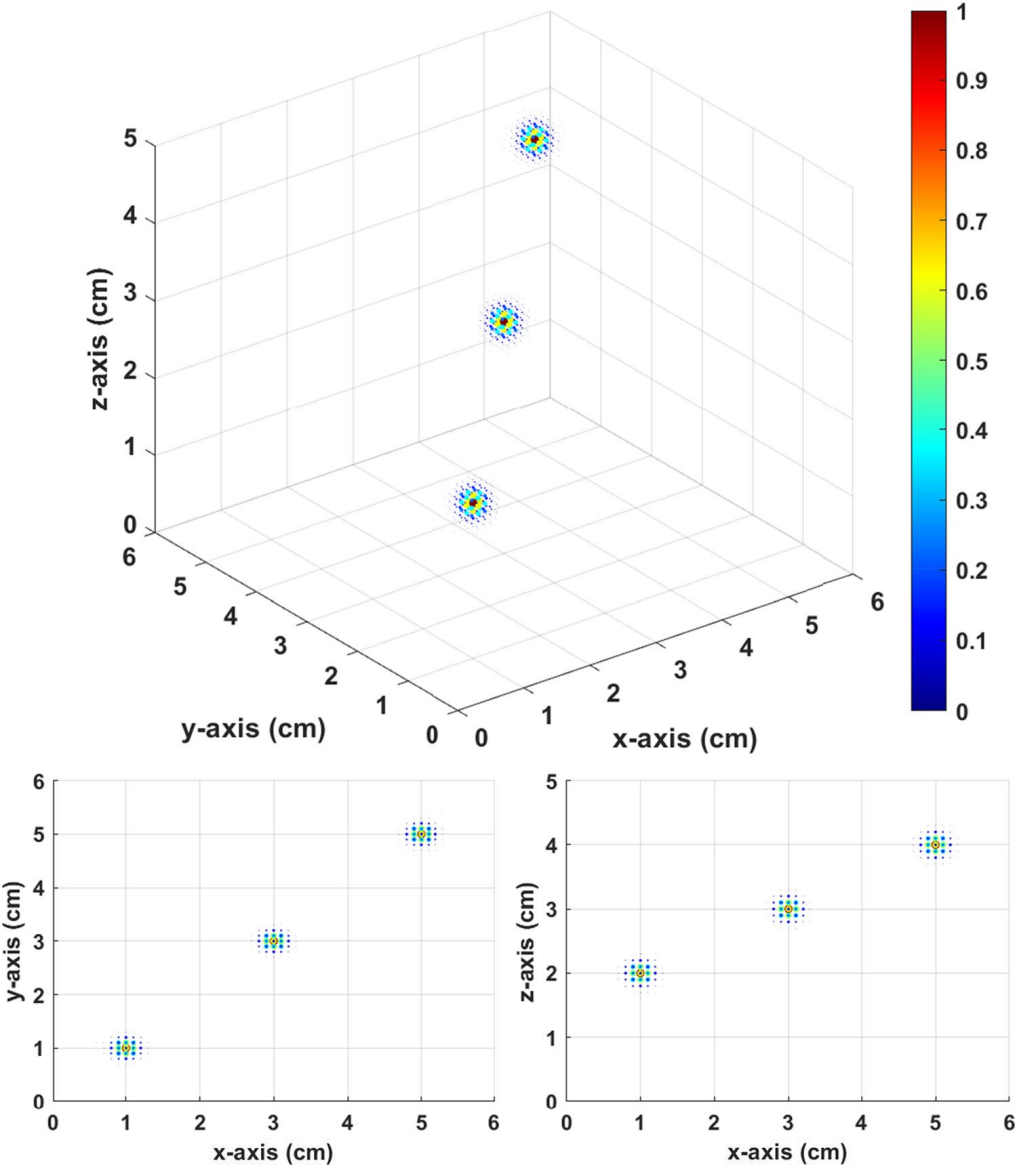
Three original sources.

The *XY* scanning plane of the sensor was 8.0 × 8.0 cm^2^ with a scanning interval of 0.5 cm. The *Z* position of the scanning plane was fixed at *Z* = 6.0 cm. The BEM was a 6.0 × 6.0 × 5.0-cm^3^ cuboid with an interval of 0.1 cm. The number of total layers was divided to 10, and the bias field was 0.5 mT. There were three setup sources at [1.0,1.0,2.0], [3.0,3.0,3.0], and [5.0,5.0,4.0], as shown in Fig. 9.

**Figure 10:**
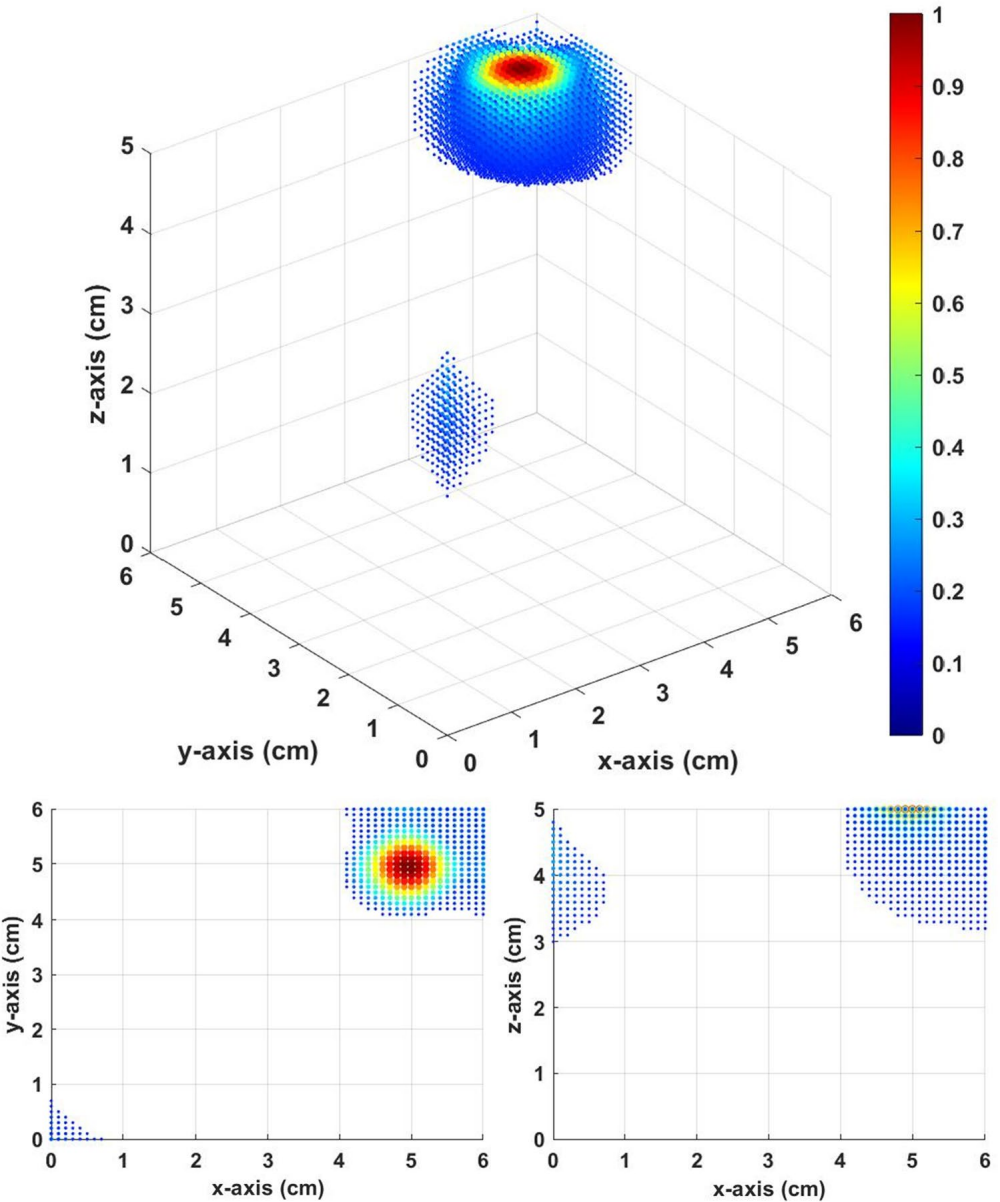
Inverse distribution from the constant field with thresholds equal to 0.15.

**Figure 11:**
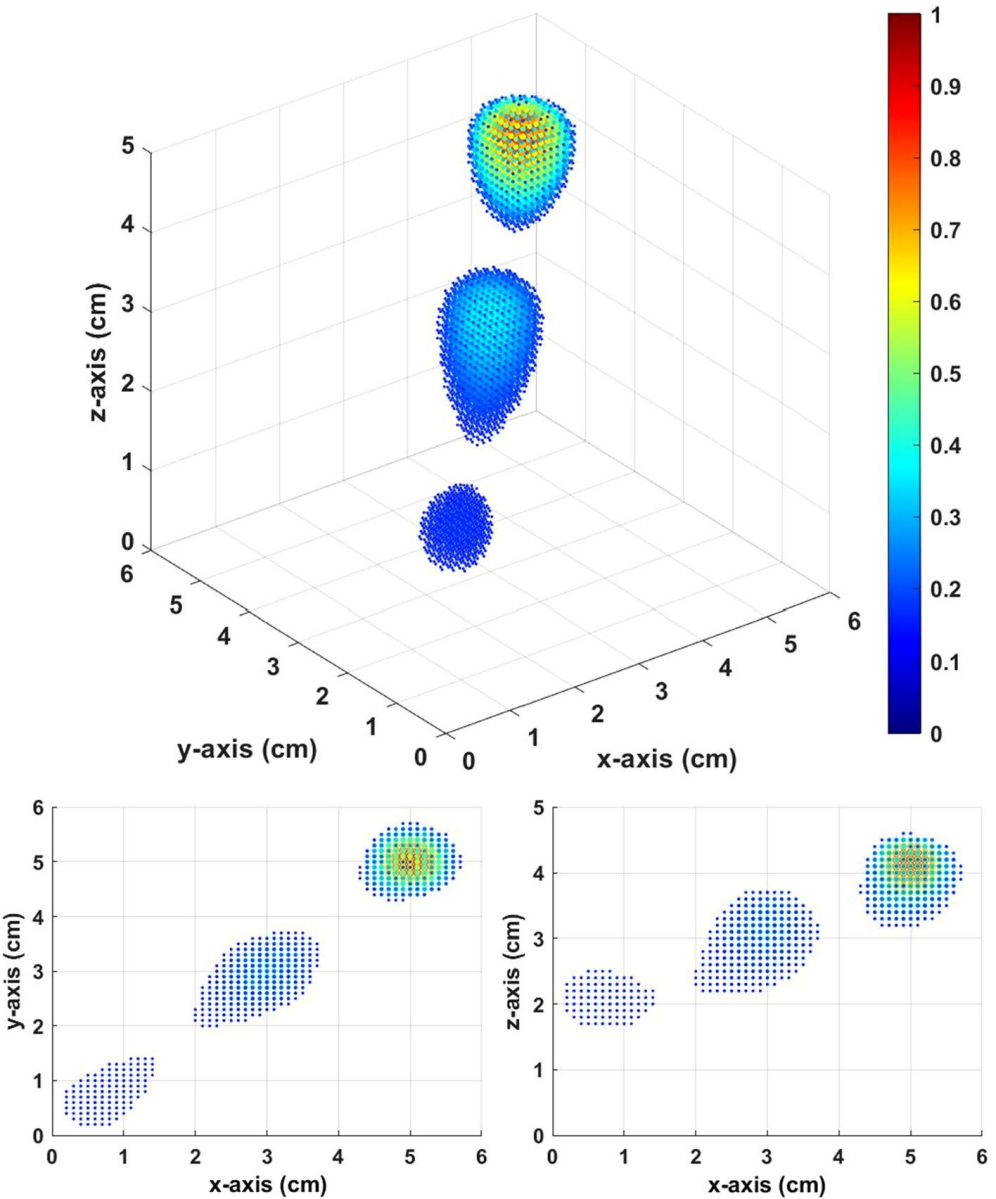
Inverse distribution from the multiple *Z* scan (gradient field) with thresholds equal to 0.15.

The inverse source distribution in Fig. 10 is calculated from the constant field. The inverse distribution is highly scattered, and we cannot distinguish the three setup sources. However, the inverse source distribution in Fig. 11 calculated from gradient field multiple *Z* scan shows a very good result. There are clearly three distinguishable local maximums in Fig. 11. The local maximums are at [0.8,0.8,2.1], [3.0,3.0,3.2], and [5.0,5.0,4.2]. The results of the gradient field multiple *Z* scan showed good accuracy in the multisource simulation.

##### Mouse brain BEM

**Figure 12:**
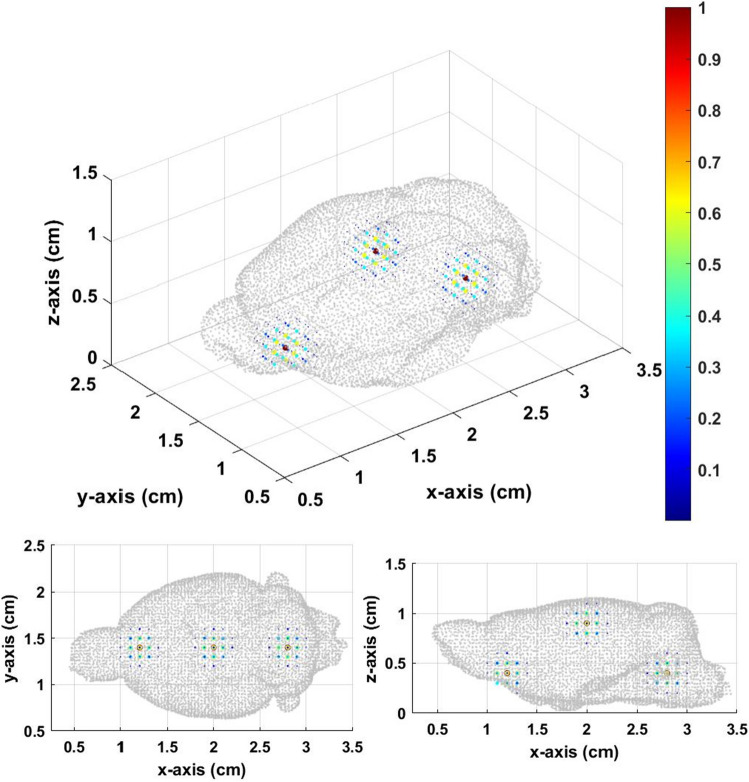
Three original sources in the mouse brain.

The experiment setup was the same as the single source in the mouse brain previously discussed. The total layer was divided to 5. There were three setup Gaussian sources centered at [1.2,1.4,0.4], [2.0,1.4,0.9], and [2.8,1.4,0.4] as shown in Fig. 12.

**Figure 13:**
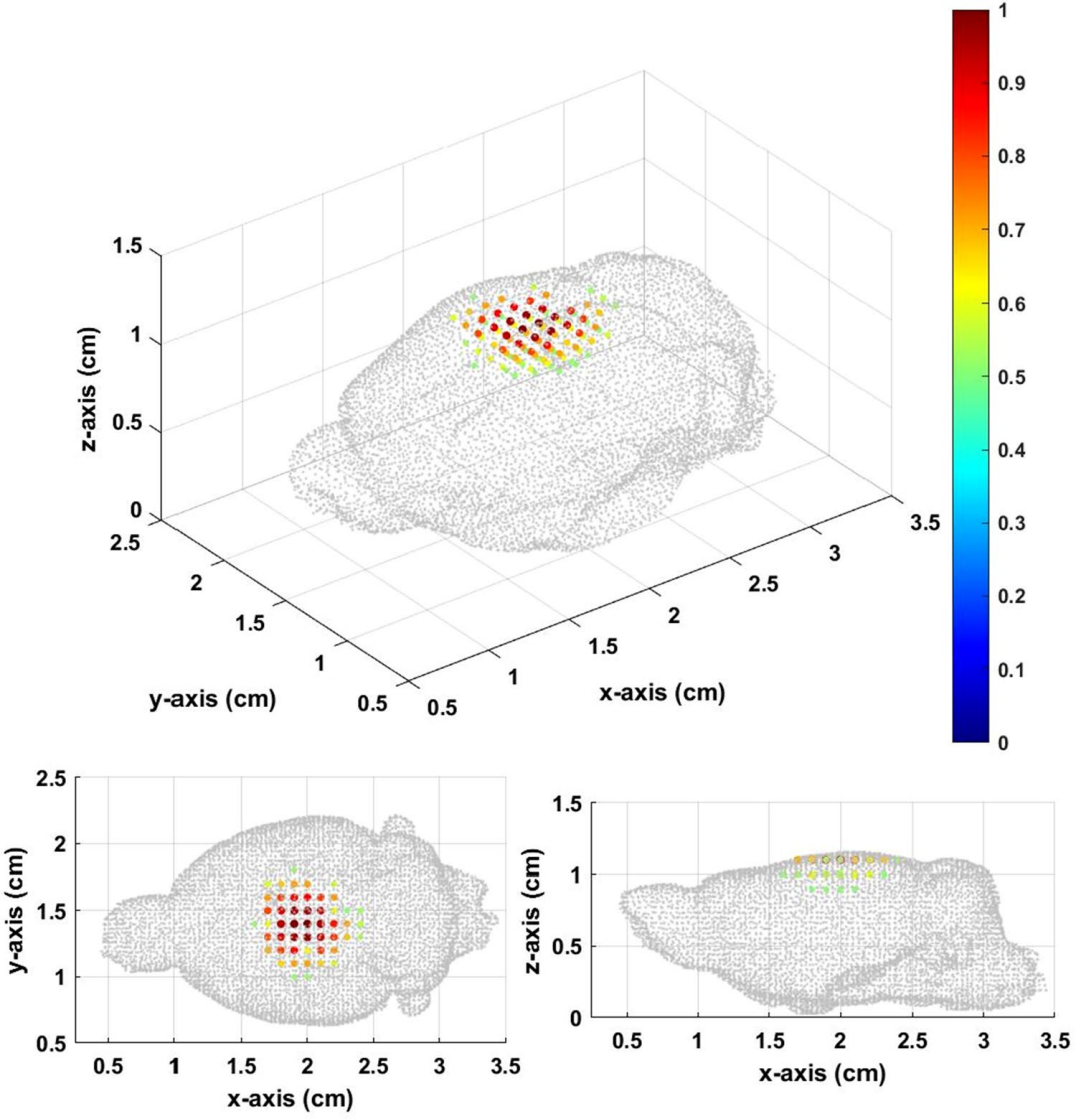
Inverse source distribution from the constant field with thresholds equal to 0.5.

**Figure 14:**
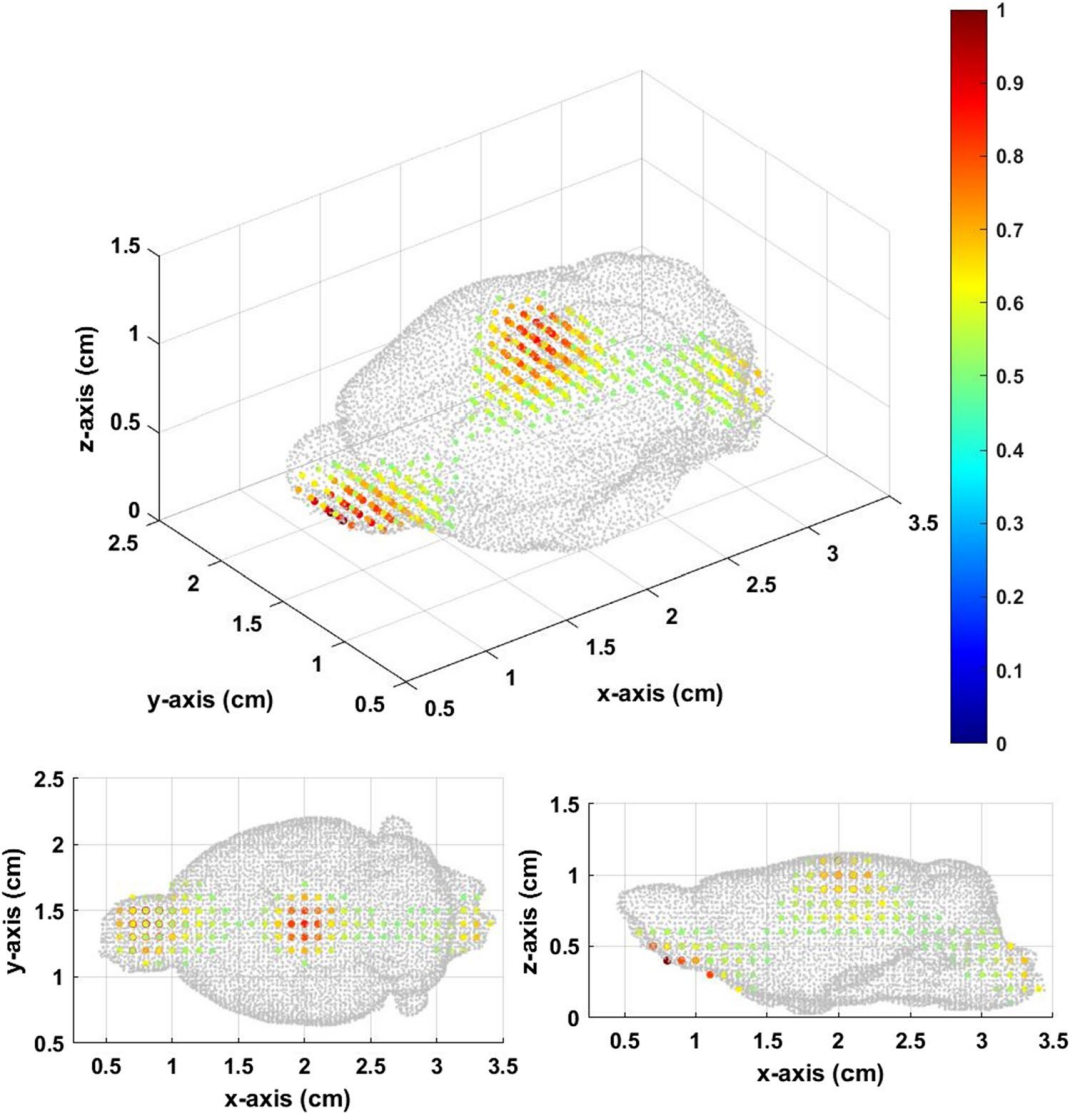
Inverse source distribution from the multiple *Z* scan (gradient field) with thresholds equal to 0.5

In Fig. 13, the inverse source distribution calculated from the constant field can only find one source at the center. We can hardly find the other two sources at the bottom. The inverse source distribution in Fig. 14 is calculated from gradient field multiple *Z* scan, which showed better results compared to that from the constant field. There are three distinguishable distributions in Fig. 14. Three local maximums are at [0.8,1.5,0.4], [2.0,1.4,1.0], and [3.3,1.3,0.4]. Although, there are some differences between the inverse and original sources, the results of the gradient field multiple *Z* scan also showed better accuracy than that of the constant field in the mouse brain.

## Conclusion

Three-dimensional forward and inverse models of were established to simulate the operation of a magnetic particle imaging (MPI) system for small animals with the linearized Bregman iterative algorithm. In this work, sensors used planar scanning to scan the target which is different from the commonly used surrounding arrangement. The spacing and range of the sensors can be adjusted arbitrarily. However, it is a time-consuming method and can be affected by noise more easily.

We focused on studying the sensors set up which had a great influence on the lead field matrix. In order to simplifier the task, we assumed that the SNR of the field maps were good enough to ignored the noise. The result showed that the farther is the sensor to the BEM, the worse is the calculated source distribution that is obtained. When the sensors moved away from the position of 2.5–6.0 cm, the location error increased from 0 to 2.449 × 10^−1^ and the average deviation increased from 2.78 × 10^−3^ to 5.88 × 10^−3^. In addition, the combination of all the scanning planes with different Z positions, a multiple scan, had almost the same inverse source distribution with the closest plane scanning at 2.5 cm. The location error and average deviation of the multiple scan are 0 and 2.84 × 10^−3^. The multiplane scanning hardly improves the accuracy of the source localization. If the noise participated in the simulation, the multiple scan could have a better result than the single scanning due to the statistical effect but the trend will still be the same.

According to Langevin function, if the magnetic field is small enough, the magnetic susceptibility is approximately linear with slope of one third. In the simulation, magnetic susceptibility of the magnetic nanoparticles (MNPs) was assumed to be a constant. The lead field matrix was differentiated to find the gradient having the maximum at the corresponding *Z* position. When the source was at the bottom of the mouse brain, the location error and average deviation of the result from the gradient scan method, multiple *Z* scan, were 2.24 × 10^−1^ and 4.42 × 10^−3^ while from the constant field were 3.61 × 10^−1^ and 5.05 × 10^−3^. The multiple *Z* scan method can improve the accuracy compared to constant field when the source is far away from the sensors. It can also better distinguish multiple sources. The multiple *Z* scan method is solid when the magnitude of the applied magnetic field does not exceed the liner zone of the magnetization curve of the MNPs. Although there is no further discussion in our simulation, the Langevin function can be substituted into the lead field matrix and the magnitude of the gradient field for having a maximum at the position we require can also be calculated by differentiation. We will conduct further research on this to make the method suitable for large applied magnetic fields in the future.

The multiple Z scan method is feasible in actual experiments, since the MPI system can add a gradient excitation field. MPI applied with constant filed is good at finding the magnetic source which is close to the sensors. The multiple Z scan method can better locate the source in deeper region. However, the magnetic field from the magnetic moment is inversely proportional to the cube of the distance approximately. The inverse distributions will both be diverse when the source is very far from the sensors. Manipulating the magnetic field and correlating with the magnetic properties of MNPs to overcome the problem caused by attenuation of the induced magnetic field have been an important research topic of the MPI. A special excitation field or MNPs with special magnetic properies could be very useful to improve the performance of the MPI. They might not need to overpower the attenuation of the response in distance. They only need to create enough deviation in the sensing matrix for algorithm to better distinguish them in a certain area.

## Methods

The basic mechanism of MPI system is described as follows: Application of external magnetic field magnetizes the magnetic particles inside the object so that the magnetic sensors can detect the induced magnetic field of the magnetic particles. The distribution of the magnetic particles is calculated by the inverse algorithm from the Field map. The detailed of the experiment of the MPI can refer to the work done by^10^.

### Forward model

As mentioned earlier, the magnetic field distribution of the magnetic source can be calculated with the forward model. The equation and corresponding meaning are shown in Eqs. (1) and (2), in which *A* represents the magnetic field operator (also known as lead field matrix), *u* represents the source distribution, and *f* represents the field map^3^.

1 $$ f = Au + k $$

where

2 $$ A = \frac{{\mu_{0} }}{4\pi }\left( {\frac{{3m_{k} \cdot \left( {r_{S,j} - r_{P,k} } \right)}}{{\|r_{S,j} - r_{P,k}\|^{5} }}\left( {r_{S,j} - r_{P,k} } \right) - \frac{{m_{k} }}{{\|r_{S,j} - r_{P,k}\|^{3} }}} \right) \cdot n_{S,j} { } $$

Equation (2) represents the induced magnetic field at the sensor position *r*_*S,j*_ generated by the magnetic moment *m*_*k*_ at *r*_*P,k*_. The *n*_*S,j*_ is the sensor direction. Subscripts *j* and *k* denote the position sequence of the sensor and grid position, respectively. With the Langevin function and external field from coil, the magnetic moment *m*_*k*_ can be obtained^4–6^. In this study, the noise was set to be zero and the magnetic susceptibility of the magnetic nanoparticles (MNPs) is a constant, i.e., the induced magnetic field of the MNPs is linear to the applied field. The forward model can calculate the field map of the source if the distribution of the source is known.

### Inverse model

The inverse problem of MPI is an ill-posed problem to find the source distribution from the magnetic field map^4–6^. The size of the operator matrix *A* depends on the sensor scanning positions and object positions. Generally, the number of object positions is much larger than the number of sensors scanning positions. The equations generally have no stable exact solutions. In other words, the inverse problem cannot be solved directly because the operator *A* is not invertible. The solution of the ill-posed problem is usually not stable, so the regularization method is applied to make the ill-posed problem become an approximated well-posed problem that can be solved properly^11^. In addition, the constraints related to our experiment can be applied to make the solution more accurate. The common regularization constraints are minimal norm, total variation, and Tikhonov regularization. The applied constraints can make the ill-posed problem solvable^11–13^. According to the references and conditions of our experiment, the constraints applied to the ill-posed problem of MPI are L1-norm regularization and positive constraint.

There are algorithms developed to find the optimal solution of the ill-posed problem such as dual ascent, dual decomposition method, alternating direction method of multipliers (ADMM), and Bregman iterative method^3,11,13–15,23^. The dual ascent method and dual decomposition method both have weak robustness. ADMM is a proper method with strong robustness based on the Lagrange multipliers method^23^. The Bregman iterative method was used in our project due to its fast and efficient feature for solving the basis pursuit problem $\min \left\{ {\left\| {{\text{u}}_{1} } \right\|:{\text{Au}} = {\text{f }},{\text{ u}} \in {\text{R}}^{{\text{n}}} } \right\}$^16,17^. The L1-norm basis pursuit problem is already applied to many fields such as image compressive^16^, multisensor networks of MRI, and CT^17–21^.

### Bregman iterative algorithm

The Bregman iterative algorithm was developed by S. Osher, M. Burger, D. Goldfarb, J. Xu, and W. Yin to solve the ill-posed problem for image processing. This method was further studied and extended to other applications of the inverse problem. The Bregman distance between points *u* and *v* is defined as follows:

$$ D_{J}^{P} \left( {u,v} \right) = J\left( u \right) - J\left( v \right) - \left\langle {P,u - v} \right\rangle $$

where *J* is a convex function mapping from a normal space *X* to a real space *R* and *P* is subgradient of *J*. There are some advantages of the Bregman distance for solving the basis pursuit problem such as the following equations:

$$ D_{J}^{P} \left( {u,v} \right) \geqq  0 \,\,\,{\text{and}}\,\,\,D_{J}^{P} \left( {v,v} \right){ } = 0. $$

The form of the problem can be generalized as $\min \left\{ {J\left( u \right) + H\left( {u,f} \right)} \right\}$.

In addition, the detail of the Bregman iterative algorithm is shown below:

Bregman iterative algorithm^16,24^:

Initialize: $$k{ }$$ = 0, $$u^{0}$$ = 0, $$P^{0}$$ = 0

While “$u^{k}$ not converge” do

$$u^{k + 1}$$ = $$\arg \mathop {{{\min}}}\limits_{u} { }D_{J}^{{P_{k} }}$$ ($${\text{u}}$$,$$u^{k}$$) + $$H$$($${\text{u}}$$)

$$P^{{{\text{k}} + 1}}$$ = $$P^{k}$$ − $$\nabla H$$($$u^{k + 1}$$)$$\in \partial J$$($$u^{k + 1}$$)

*k* = *k* + 1

end while

if *H*(*u*) = $$\frac{1}{2}\left\| {Au - f} \right\|_{2}^{2} { }\& J\left( u \right) = \left\| u \right\|_{1}$$

The equation becomes the familiar form of the basis pursuit problem:

$$ \mathop {\min }\limits_{{\text{u}}} \frac{1}{2}\left\| {Au - f} \right\|_{2}^{2}  + \left\| u \right\|_{1}   $$

The Bregman algorithm requires to find minimization at each step of the algorithm. We simplified the calculation by linearizing the algorithm. Besides, a penalty term can be added due to the limitation of the linear approximation. The equation is now written as follows:

$$ u^{k + 1} = \arg \mathop {\min}\limits_{{\text{u}}} D_{J}^{{P^{k} }} \left( {u,u^{k} } \right) + H\left( {u^{k} } \right) + \left\langle {\nabla H\left( {u^{k} } \right),u - u^{k} } \right\rangle + \frac{1}{2\delta }\left\| {u - u^{k} } \right\|_{2}^{2} $$

With substitution of

$$ H\left( u \right) = \frac{1}{2}\left\| {Au - f} \right\|_{2}^{2} { },{ }J\left( u \right) = \left\| u \right\|_{1} $$

and some calculations, the following equation can be obtained:

$$ u^{k + 1} = \mathop {{{\min}}}\limits_{u} \mathop \sum \limits_{i = 1}^{n} \left| {u_{i} } \right| - \left\langle {u,v^{k - 1} - \frac{{u^{k} }}{\delta }} \right\rangle + \frac{1}{2\delta }\left\| {u - \left( {u^{k} - \delta A^{T} \left( {Au^{k} - f} \right)} \right)} \right\|_{2}^{2} + C. $$

*u* is component independent, so the minimization problem can be solved separately.

The shrinkage function is shown as follows:

$$ u_{i}^{k + 1} = \delta \left( {v_{i}^{k} - \mu } \right)\quad v_{i}^{k} \in \left( {\mu ,\infty } \right) $$

$$ u_{i}^{k + 1} = 0\quad v_{i}^{k} \in \left( { - \mu ,\mu } \right) $$

$$ u_{i}^{k + 1} = \delta \left( {v_{i}^{k} + \mu } \right)\quad v_{i}^{k} \in \left( { - \infty ,\mu } \right). $$

Linearized Bregman algorithm^16,24^:

Initialize: $$u$$ = 0, $$v$$ = 0

while “||*f* – *A* × *u*|| does not converge” do

$$v^{k + 1} = { }v^{k}$$ + $${\text{A}}^{{\text{T}}}$$ ($${\text{f}} - {\text{Au}}^{{\text{k}}}$$)

*u*^*k*+1^ $$= { }\delta {\text{shrink}}\left( {v^{k + 1} } \right)$$

end while

The basis pursuit problem can be solved by the iterative method. Now, the inverse model of MPI has been established and it can be used to solve the source distribution from the field map.

The initial value of the source distribution *u* is set to zero, and the residual is defined by norm (field map − LF × *u*)/norm (field map). The value of the residual can help to determine the degree of data fitting at every algorithm step. The algorithm will keep running until the difference between the residual of this step and the last step is smaller than 10^−6^.

### Experiment procedure

The BEM grids to construct the calculation space were first set up. The sensor scanning mode was single or multiple rectangular *XY* planes above the BEM. The size of the scanning plane was 1 cm larger than the maximum edge of the BEM in the *XY* directions. After the BEM and sensor positions were set up, the forward model was established to calculate the field map with the set-up source distribution. Afterwards, the Bregman iterative algorithm was applied to solve the source estimation problem from the field map. The “Location error” and “Average deviation” were defined to evaluate the conformity of the inverse and original source distributions^22^.

3 $$ {\text{Average deviation}} = \frac{{\Sigma \left| {D_{{{\text{inv}}}} - D_{{{\text{ori}}}} } \right|}}{N} $$

4 $$ {\text{Location}}\,\,{\text{error}} = {\text{norm}}({\text{Location}}_{{{\text{orig}}}} - {\text{Location}}_{{{\text{inv}}}} ) $$

### Gradient scan method

The simulation results above showed that multilayer scanning does not effectively improve the calculation accuracy of the source, especially for the deeper sources. Therefore, a new method to improve the accuracy of source distribution was developed in this work.

Lead field matrix depends on the spatial structure and external field. The spatial structure is based on the actual experimental setup and cannot be changed. Therefore, the gradient field was applied instead of the constant field to modify the magnitude of the lead field matrix. The gradient field can adjust the weights of lead field matrix which correspond to different positions of the BEM.

Generally, it is a challenge for MPI system to measure a magnetic source which is far from the sensor because the signal strength decreases drastically with the distance. According to our experimental setup, the scanning plane of the sensor was set at the *XY* plane. The distance in the equation of the forward operator *A* is three dimensional. Our main purpose was to improve the accuracy of source distribution along the *Z* direction. Gradient field was applied along the *Z* axis to modify the lead field matrix so that source at deeper position *Z* could have a larger response.

5 $$ B\left( {r_{P,z} } \right) = {\text{ gradient}} \times r_{P,z} + {\text{bias}} $$

In order to have the largest response among others along the *Z* axis (gradient field to scan), the forward operator *A* was differentiated with respect to the *Z* position of the BEM $r_{P,z}$ and required

6 $$ \frac{{{\text{d}}A_{Z} \left( {r_{P,z} ,B\left( {r_{P,z} } \right)} \right)}}{{{\text{d}}r_{P,z} }} = 0. $$

The bias field was fixed to calculate the gradient from the formula obtained by differential equation. The gradient that should be applied was determined in order for lead field matrix to have local maximum at a certain layer with a certain *Z* coordinate of the BEM. Then, all the lead field matrices with different gradient fields that made lead field matrix to have local maximum at the different layers of the *Z* coordinate into a single lead field matrix were combined. The method that was developed in this work used gradient field to scan the BEM instead of actual sensor scanning from a certain point of view.

**Figure 15:**
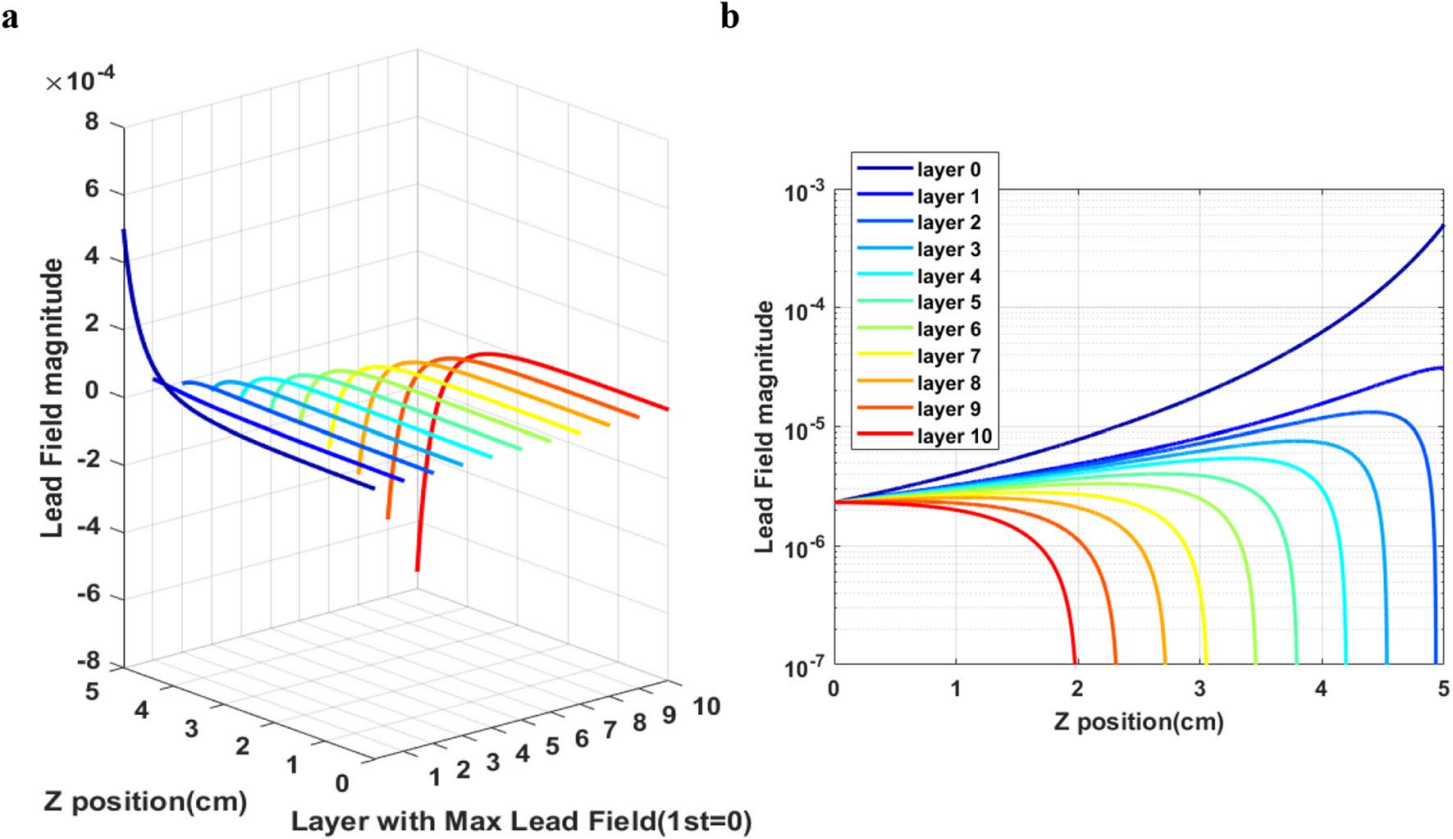
(**a**) Lead field magnitude with maximum at different *Z* layers and (**b**) lead field magnitude with maximum at different *Z* layers in two-dimensional view.

In Fig. 15, ten layers of different *Z* positions of the BEM were selected. The differential equation was calculated to find the gradient that made the lead field matrix to have local maximum at the corresponding layer. The BEM used here measured 6.0 × 6.0 × 5.0 cm^3^, the *Z* position of the sensor was at 6.0 cm, and the uniform bias field was 0.5 mT. Layer 0 represents the constant field without gradient. The lead field magnitude increased as the BEM position became closer to the sensor when the applied field was a constant field. The lead field magnitude has a local maximum at the selected layer from top to bottom of the BEM. The layer with small *Z* position was far from the sensor, and the local maximum caused by gradient field involved the change of sign of value.

### Mouse brain model

Magnetic Resonance Imaging (MRI) images were obtained by the Bruker 7 T MRI Biospec 70/30 with the resolution of 128*256*384 (100 um^3^) and 3D gradient echo sequence. The MRI images of mouse brain were applied to the open source software Brainsuite to create the BEM model.

### Approval of animal use

Animal use was approved by National Taiwan University College of Medicine and College of Public Health Institutional Animal Care and Use Committee (IACUC) with the IACUC Approval Number 20170556. And all experiments in this study were performed in accordance with relevant guidelines and regulations.
